# Supplementary material for: Microbiota and Serum Metabolic Profile Changes in Korean Native Hanwoo Steer in Response to Diet Feeding Systems
Source: Int J Mol Sci. 2022 Oct 16;23(20):12391. doi: 10.3390/ijms232012391 (PMC9604148; doi:10.3390/ijms232012391)
Supplement: Supplementary file 1 [file ijms-23-12391-s001.zip › ijms-1902011-supplementary.pdf]

**Microbiota and Serum Metabolic Profile Changes in Korean Native Hanwoo Steer in Response to Diets Feeding System**

Jeong Sung Jung [1], Ilavenil Soundharrajan [1], Dahye Kim [2], Myunggi Baik [3], Seungmin Ha [4] and Ki Choon Choi [1,*]

1 Grassland and Forage Division, National Institute of Animal Science, Rural Development Administration, Cheonan 31000, Korea
2 Animal Genomics and Bioinformatics Division, National Institute of Animal Science, Wanju 55365, Korea
3 Department of Agricultural Biotechnology and Research Institute of Agriculture and Life Sciences, College of Agriculture and Life Sciences, Seoul National University, Seoul 08826, Korea
4 Dairy Science Division, National Institute of Animal Science, Rural Development Administration, Cheonan 31000, Korea
* Correspondence: choiwh@korea.kr; Tel.: +82-41-580-6752; Fax: +82-41-580-6779

**Supplementary Table S1.** Effects of feeding systems on Grazing on growth performance and feed intake in Hanwoo steers during growing period.

| **Items** | **Total period** | | | |
|---|---|---|---|---|
| | **Control (Housing)** | **Grazing** | **SEM** | **Pr>F** |
| Initial body weight (kg) | 260.80 | 219.80 | 14.70 | 0.16 |
| Final body weight (kg) | 376.40 | 415.00 | 11.50 | 0.26 |
| Average daily gain (ADG, kg) | 0.81 | 0.83 | 0.03 | 0.22 |
| Feed intake (kg) | 7.80 | 8.22 | 0.20 | 0.09 |
| Concentrate (kg) | 3.95 | 3.03 | 0.18 | 0.01 |
| Rice straw (kg) | 3.85 | - | 0.07 | - |
| Grass/grazing (kg) | - | 5.19 | 0.22 | - |
| Feed efficiency | 0.11 | 0.09 | 0.01 | 0.03 |
| Feed conversion ration | 9.63 | 11.10 | 0.62 | 0.02 |
| Intake(kg) | | | | |
| Crude protein | 152.14 | 282.82 | 22.48 | 0.01 |
| Total digestibility nutrients ( TDN) | 932.87 | 1,173.05 | 46.94 | 0.01 |

SEM: Standard error of Mean

**Supplementary Table S2.** Ingredient composition and chemical analysis of nutrients.

| Items | Compositions (% DM) |
|---|---|
| ***Ingredients*** | |
| Cornflake | 25 |
| Wheat | 18 |
| Gluten feed | 8 |
| Tapioca residue | 4.58 |
| Wheat bran | 11.79 |
| Palm kernel meal | 8 |
| Coconut oil meal | 3 |
| Rapseed meal | 5 |
| Soybean meal | 7.39 |
| disillers dried grains | 2.22 |
| Limestone | 2.17 |
| Molasses | 3 |
| Salt | 0.81 |
| Probiotics | 0.05 |
| Magnesium oxide | 0.3 |
| Sodium bicarbonate | 0.3 |
| Vitamin premix[a] | 0.21 |
| Mineral premix[b] | 0.18 |
| Total | 100 |
| ***Chemical compositions*** | |
| Dry matter (DM) | 88.23 |
| Crude protein (CP) | 16.36 |
| Ether extract | 11.25 |
| Crude fiber | 26.78 |
| Acid detergent fiber (ADF) | 22.5 |
| Neutral detergent fiber (NDF) | 35.50 |
| Total digestible nutrient (TDN) | 74.68 |

[a]Vitamin premix contained the following ingredients diluted in cellulose (g/kg premix): L-ascorbic acid, 121.2; DL-α-tocopherol acetate, 18.8; thiamine hydrochloride, 2.7; riboflavin, 9.1; pyridoxine hydrochloride, 1.8; niacin, 36.4; Ca-D-pantothenate, 12.7; myoinositol, 181.8; D-biotin, 0.27; folic acid, 0.68; p-aminobenzoic acid, 18.2; menadione, 1.8; retinal acetate, 0.73; cholecalciferol, 0.003; cyanocobalamin, 0.003. [b]Mineral premix contained the following ingredients (g/kg premix): Mg $SO_4$_$7H_2O$, 80.0; $NaH_2PO_4$ $2H_2O$, 370.0; KCl, 130.0; ferric citrate, 40.0; $ZnSO_4$_$7H_2O$, 20.0; Ca-lactate, 356.5; CuCl, 0.2; $AlCl_3$_$6H_2O$. 0.15; KI, 0.15; $Na_2Se2O_3$, 0.01; $MnSO_4$_$H_2O$, 2.0; $CoCl_2$_$6H_2O$, 1.0. Concentrate feed was provided by NongHyup Company. [c]Vitamin premix contained the following ingredients (Power Vitamine, Genobio, Republic of Korea) : Vitamin A, 6,000,000IU; Vitamin D3, 1,200,000 IU; Vitamin E, 1,000mg; Vitamin B1, 500mg; Vitamin B2,

500mg; Vitamin B6, 500mg; Vitamin B12, 10mg; Protected Vitamin C, 5,000mg; Pantothenic acid, 1,000mg; Niacin, 1,000mg; Biotin, 30mg; Folic acid, 600mg; Mn, 100mg.

**Supplementary Table S3.** Nutrient compositions of rice straw and grass during growing period

| Item (% DM) | Rice straw | Grass/Grazing | | | | | | |
|---|---|---|---|---|---|---|---|---|
| | | May | Jun. | Jul. | Aug. | Sep. | Oct. | Nov |
| DM | 88.82±0.36 | 22.50±2.04 | 30.00±4.02 | 34.66±2.94 | 31.32±0.17 | 27.32±0.82 | 29.73±0.29 | 30.02±0.44 |
| CP | 4.45±0.02 | 12.37±0.47 | 12.05±1.08 | 14.51±0.04 | 13.57±0.47 | 18.60±0.27 | 15.37±0.42 | 15.63±0.49 |
| ADF | 42.20±0.22 | 30.61±0.70 | 39.47±1.06 | 40.24±0.14 | 41.48±0.58 | 38.43±0.49 | 35.07±0.39 | 32.95±0.79 |
| NDF | 68.99±0.08 | 54.22±0.04 | 64.32±0.72 | 61.29±1.24 | 60.57±0.85 | 60.04±0.87 | 57.26±0.36 | 55.68±0.72 |
| TDN | 55.56±0.17 | 64.72±0.55 | 57.72±0.84 | 57.11±0.11 | 56.13±0.46 | 58.54±0.39 | 61.19±0.31 | 62.87±0.63 |
| IVDMD | 40.07±0.87 | 75.58±1.28 | 70.62±2.38 | 71.19±0.61 | 71.39±0.56 | 73.18±0.65 | 74.56±0.57 | 74.70±0.74 |

DM: Dry matter, CP: crude protein, AFD: Acid detergent fiber , NDF: Neutral detergent fiber, TDN: Total digestible nutrient, IVDMD: In vitro dry matter digestibility

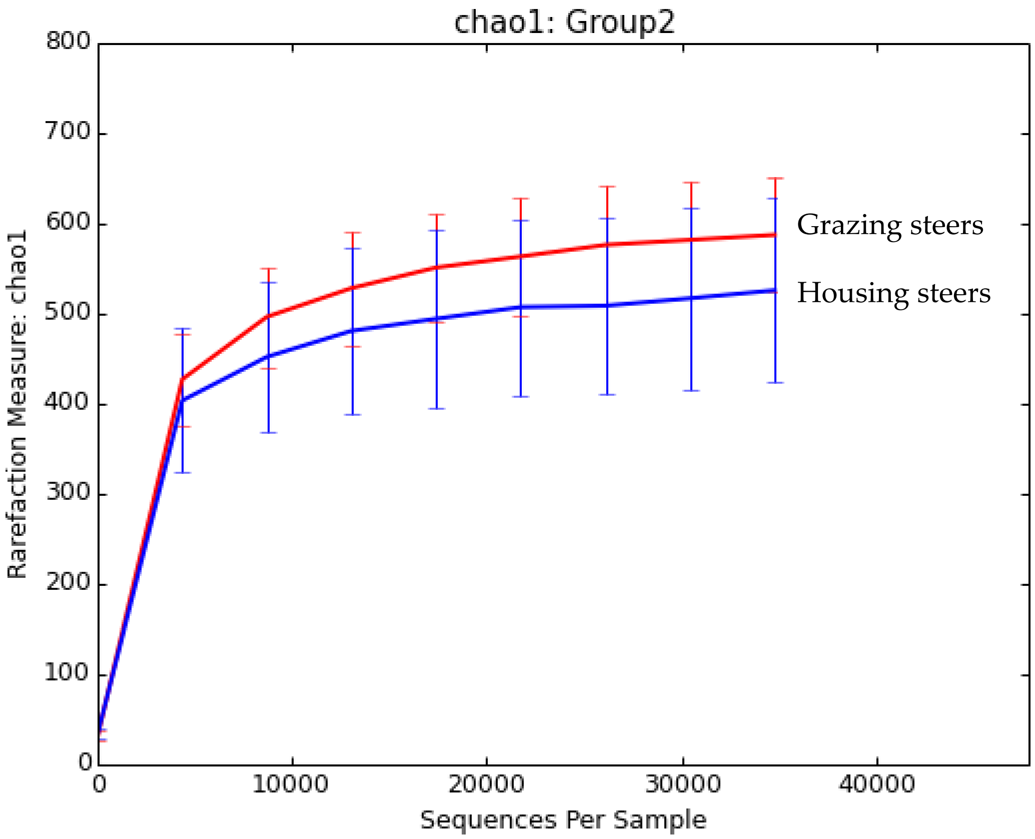


**Supplementary Figure S1.** A rarefaction analysis of the fecal microbiota of grazing and housing steers
